# Expanding the Genetic Code for Neuronal Studies

**DOI:** 10.1002/cbic.202000300

**Published:** 2020-07-20

**Authors:** Ivana Nikić‐Spiegel

**Affiliations:** ^1^ Werner Reichardt Centre for Integrative Neuroscience University of Tübingen Otfried-Müller-Strasse 25 72076 Tübingen Germany

**Keywords:** genetic code expansion, mutagenesis, neurobiology, protein engineering, unnatural amino acids

## Abstract

Genetic code expansion is one of the most powerful technologies in protein engineering. In addition to the 20 canonical amino acids, the expanded genetic code is supplemented by unnatural amino acids, which have artificial side chains that can be introduced into target proteins in vitro and in vivo. A wide range of chemical groups have been incorporated co‐translationally into proteins in single cells and multicellular organisms by using genetic code expansion. Incorporated unnatural amino acids have been used for novel structure‐function relationship studies, bioorthogonal labelling of proteins in cellulo for microscopy and in vivo for tissue‐specific proteomics, the introduction of post‐translational modifications and optical control of protein function, to name a few examples. In this Minireview, the development of genetic code expansion technology is briefly introduced, then its applications in neurobiology are discussed, with a focus on studies using mammalian cells and mice as model organisms.

## Graphical abstract

**Do what comes unnaturally**: The genetic code has been expanded to include unnatural amino acids (UAAs). Thanks to their unique side chains, UAAs confer new properties on target proteins. These allow us to modify biological processes with unprecedented molecular precision. The applications of this technology in neurobiology are the focus of this Minireview.

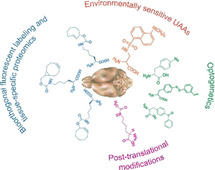

## 1 Introduction

Proteins play central roles in biological processes. Accordingly, techniques for visualising and modifying proteins have driven important discoveries in neuroscience, and indeed, the life sciences in general. For example, fluorescent proteins are used to label cells, organelles and proteins of interest in healthy and pathological nervous systems.^[1]^ Fluorescent proteins can also be used as biosensors, for example, for studying synaptic transmission, membrane voltage and redox status.^[2]^ Photosensitive proteins are used in optogenetics for controlling neuronal activity.^[3]^ All of these examples are based on protein engineering, and rely either on directing exogenous proteins, such as light‐sensitive opsins or biosensors, to the cells of interest, or on making chimeric fluorescent protein fusions. Although powerful, these approaches do not allow the engineering of target proteins at the submolecular level.

To obtain that submolecular resolution, proteins should be modified at the level of their building blocks, that is, the amino acids. This is, to some extent, possible with mutagenesis. However, because the genetic code relies on a fixed number of standard amino acids, conventional mutagenesis is limited in scope. This limitation can be overcome by expanding the genetic code to utilise unnatural amino acids (UAAs).^[4]^ UAAs, also referred to as noncanonical (or non‐natural) amino acids, carry artificial side chains that can confer new chemical and physical properties on proteins in vitro and in vivo. UAAs can be incorporated in vitro by different synthetic, semisynthetic and biosynthetic methods, such as native chemical ligation, intein‐mediated protein ligation, protein *trans*‐splicing, cell‐free and flexible in vitro translation.^[5]^ Genetic code expansion (GCE) enables the incorporation of UAAs into proteins in bacteria, yeast, cell lines, multicellular organisms, and human hematopoietic stem cells.[^4c^, ^4d^, ^6^] This affords us the opportunity to modify and visualise dynamic biological processes with unprecedented submolecular precision. The application of this emerging technology in neurobiology is the focus of this Minireview.

## 2 The Development of Genetic Code Expansion Technology

Protein translation relies on the pool of 20 standard canonical amino acids that are used by organisms across the three domains of life (Bacteria, Archaea, Eukarya). In addition, some organisms use one or both of two rare proteinogenic amino acids: selenocysteine, a 21st amino acid found in all three domains of life^[7]^ and pyrrolysine (Pyl), a 22nd amino acid found in some methanogenic archaea and bacteria.^[8]^ Thanks to the developments in chemical and synthetic biology, this pool of 22 amino acids is widely supplemented by UAAs.

**Figure 1:**
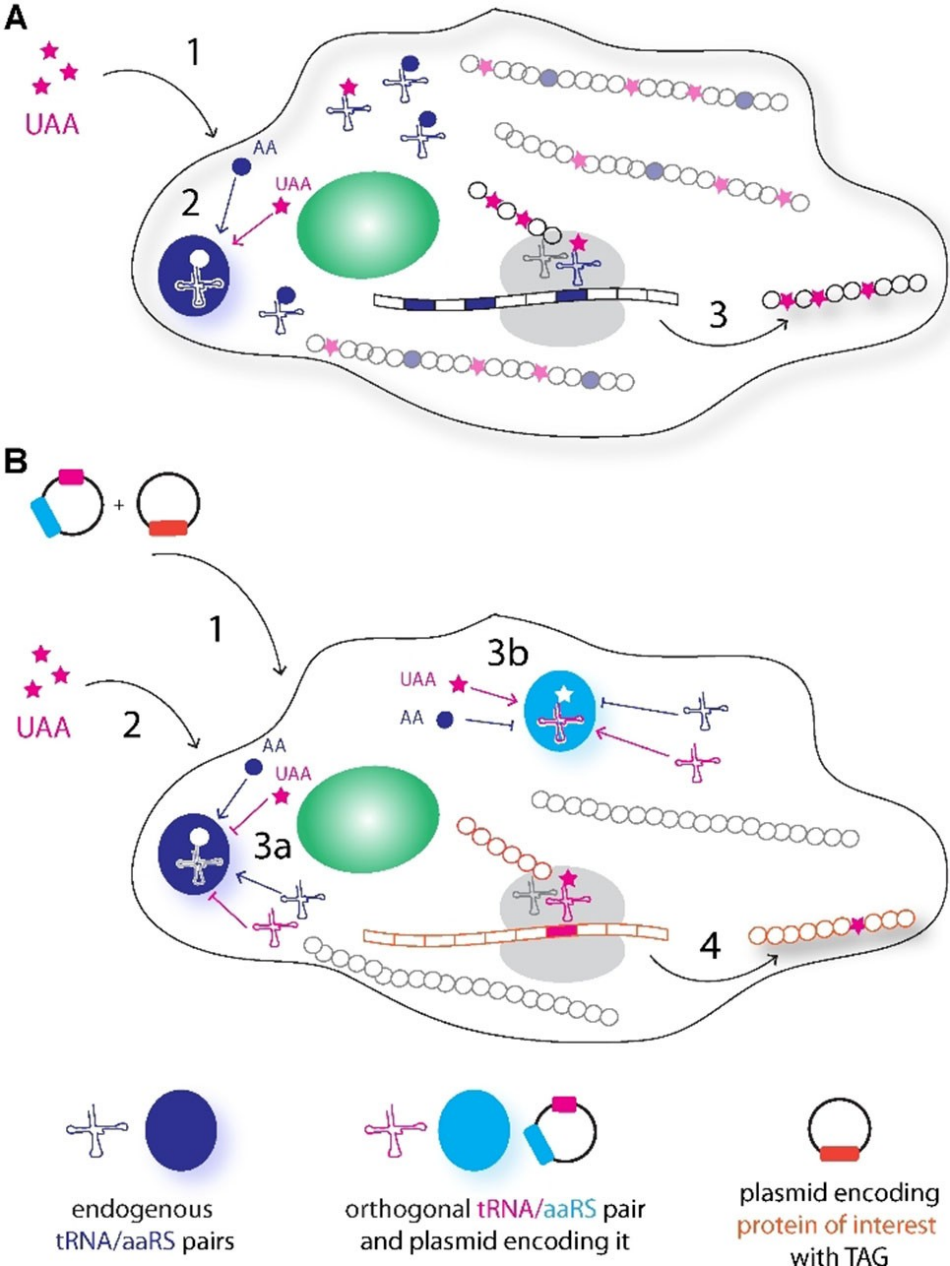
Comparison of A) UAA‐based metabolic labelling and B) genetic code expansion (GCE) technology. During metabolic labelling, UAAs are incorporated in a residue‐specific way by competing with canonical amino acids for access to the native translational machinery. The following steps are depicted: 1) addition of UAA to the medium; 2) competition between AA and UAA for the same aaRS; 3) proteome‐wide incorporation of the UAA. In contrast, genetic code expansion (B) allows site‐specific incorporation of UAAs only in the protein of interest by using orthogonal tRNA/aaRS pairs. The following steps are depicted: 1) transfection with plasmids containing orthogonal tRNA/aaRS pairs and proteins of interest with the amber codon; 2) addition of UAA to the medium; 3a) orthogonality: endogenous aaRS does not accept orthogonal tRNA and UAA; 3b) orthogonality: UAA is accepted only by the orthogonal tRNA/aaRS pair; 4) site‐specific incorporation of the UAA only in the protein of interest.

UAAs can be incorporated into proteins inside cells in two ways. One option is metabolic incorporation by the native translation machinery (Figure 1A).^[9]^ During metabolic labelling, a UAA replaces one of the natural amino acids and is incorporated proteome‐wide in a residue‐specific way. A main disadvantage of this approach is the competition between the UAA and the canonical amino acid for the components of the native translational machinery. The second option avoids this problem by equipping the host cell with an orthogonal translational machinery. In this case, UAAs are incorporated site‐specifically into one target protein without competition from any of the canonical amino acids. This is achieved by GCE (Figure 1B).

Similar to canonical amino acids, genetic encoding of UAAs requires a set of components that translate information from mRNA codons into the correct amino acid sequence during protein synthesis: 1) an orthogonal aminoacyl‐tRNA synthetase (aaRS)/tRNA pair that is specific for the UAA; 2) a unique codon to which the orthogonal tRNA will bind during protein translation; 3) a UAA that can be delivered efficiently to the host cell (Figure 1B).

To maintain the fidelity of translation and ensure specific incorporation of the UAA, orthogonality of aaRS/tRNA pairs is a prerequisite. It ensures that an imported aaRS will not aminoacylate any of the host tRNAs and that host aaRSs will not aminoacylate imported tRNA. This is best achieved by importing tRNAs and aaRSs from other domains of life. In the case of cross‐species aminoacylation, the imported tRNA/aaRS pair needs to be further evolved to be orthogonal in the host cell. In addition, it needs to be made specific for the desired UAA. This is usually achieved by successive rounds of aaRS/tRNA engineering and selection.^[10]^ Recently, a large‐scale screening method based on genomic data and tRNA identity element scoring was developed and applied to rapidly identify new orthogonal aaRS/tRNA pairs.^[11]^ To avoid competition with native tRNAs and to allow site‐specific incorporation of the UAA, orthogonal tRNAs need to recognise a unique, blank codon. In this regard, stop (also called nonsense) codons are considered unique because (with few exceptions) they do not encode any of the canonical amino acids. Typically, the amber stop codon (UAG) is reassigned for incorporation of UAAs because of its lowest abundance in both prokaryotes and eukaryotes. Accordingly, the method is known as amber codon suppression. Other stop codons (opal and ochre) or quadruplet codons can also be used.^[12]^ Before GCE was developed, UAAs were incorporated inside cells, primarily *Xenopus* oocytes, by microinjection of the mutant mRNA and chemically aminoacylated tRNA.^[13]^ This allowed for molecular studies of many neuronal proteins, such as ion channels and neuroreceptors.[^5g^, ^14^] However, this approach is suboptimal because the chemically aminoacylated tRNAs cannot be recycled during protein synthesis, leading to low expression efficiency. For that reason, considerable effort has been put into genetically encoding orthogonal aaRS/tRNA pairs.

**Figure 2:**
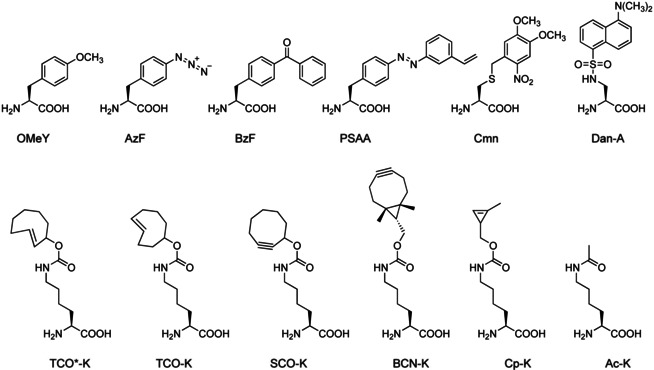
Structures of UAAs discussed in this Minireview. *O*‐Methyl‐l‐tyrosine (OMeY); *p*‐azido‐l‐phenylalanine (AzF); *p*‐benzoyl‐l‐phenylalanine (BzF, also known as Bpa); azobenzene‐based photoswitchable UAA (PSAA); 4,5‐dimethoxy‐2‐nitrobenzyl‐cysteine (Cmn), dansyl‐alanine (Dan‐A); *trans*‐cyclooct‐2‐en‐lysine (TCO*‐K); *trans*‐cyclooct‐4‐en‐lysine (TCO−K); strained cyclooctyne‐lysine (SCO−K); bicyclononyne‐lysine (BCN−K); cyclopropene‐lysine (Cp−K); *N*‐acetyl‐lysine (Ac−K).

A genetic code was first expanded in *Escherichia coli* in 2001 by using the tyrosyl‐RS/tRNA^Tyr^ pair from the archaebacterium *Methanocaldococcus jannaschii* (formerly *Methanococcus jannaschii*).^[15]^ Wang et al. mutated *M. jannaschii* tRNA^Tyr^ to recognise the amber codon, improved its orthogonality by a selection from a random *M. jannaschii* tRNA library and tuned the substrate specificity of *M. jannaschii* TyrRS to incorporate *O*‐methyl‐l‐tyrosine (OMeY, Figure 2) into a protein in *E. coli*.

Later, the substrate specificity of the same synthetase was further evolved to process other UAAs. However, the *M. jannaschii* TyrRS/tRNA pair is orthogonal in *E. coli*, but not in eukaryotes. To expand the genetic code of eukaryotes, aaRS/tRNA pairs from *E. coli* were evolved. These include TyrRS/tRNA^Tyr^, LeuRS/tRNA^Leu^ and TrpRS/tRNA^Trp^ pairs.^[16]^ Another important system for GCE is the PylRS/tRNA^Pyl^ pair.[^8a^, ^8b^, ^17^] Owing to the unique structural features of the PylRS/tRNA^Pyl^ pair, it is orthogonal in both prokaryotes and eukaryotes.[^8a^, ^18^] This makes it possible to fine tune and evolve PylRS variants in *E. coli* before transferring them to more complex systems, such as eukaryotic cells.^[19]^ Owing to its substrate promiscuity, PylRS can be used to incorporate a large number of diverse UAAs. Two systems are widely used: *Methanosarcina mazei* PylRS/tRNA^Pyl^ (*Mm* PylRS/tRNA^Pyl^) and *Methanosarcina barkeri* PylRS/tRNA^Pyl^ (*Mb* PylRS/tRNA^Pyl^). Recently, mutually orthogonal PylRS/tRNA^Pyl^ pairs were reported.^[20]^ In combination with the *Mm* PylRS/tRNA^Pyl^ pair, they can be used to genetically encode two distinct UAAs in response to two different codons in proteins in *E. coli* and mammalian cells.[^20a^, ^20b^, ^20c^] Furthermore, with triply orthogonal aaRS/tRNA pairs, it is even possible to incorporate three distinct UAAs in a single target protein in *E. coli*.^[20d]^

### 2.1 Genetic code expansion in mammalian cells

After the breakthrough in *E. coli* was achieved, the next milestone was transferring that success to eukaryotes. It had been shown that the *E. coli* TyrRS/tRNA^Tyr^ amber suppression pair is orthogonal in yeast,^[21]^ but the first study in which this pair was used to incorporate UAAs in yeast reported low UAA incorporation efficiency.^[16a]^ Subsequent attempts to establish a more robust GCE in eukaryotes were done in parallel for yeast and mammalian cells, but because the focus of this Minireview is neurobiology, only the mammalian studies are discussed.

One of the main challenges of GCE in mammalian cells, and eukaryotes in general, is the difference between tRNA transcription in bacteria and eukaryotes. The use of potentially orthogonal *E. coli* aaRS/tRNA pairs in mammalian cells therefore represents a significant challenge. In contrast to eukaryotes, bacteria have only one polymerase that transcribes all RNA molecules, including tRNAs. Bacterial tRNA promoters are located upstream of the tRNA gene. By contrast, eukaryotes have three RNA polymerases – pol I, pol II and pol III – relying on pol III for tRNA transcription. Pol III recognises intragenic promoter elements, known as A‐ and B‐boxes, within eukaryotic tRNA genes.^[22]^ For that reason, simply inserting bacterial tRNAs into mammalian cells cannot work. Attempts to introduce A‐ and B‐boxes into orthogonal tRNA genes were successful in yeast, but led to nonfunctional tRNAs in mammalian cells.^[23]^ As a result, a different approach has had to be developed for GCE in mammalian cells.

One strategy for overcoming this problem involved a special type of prokaryotic tRNA^Tyr^ from *Geobacillus stearothermophilus* (formerly *Bacillus stearothermophilus*). This tRNA differs from other bacterial tRNAs in the sense that it contains internal promoter sequences and can be expressed in mammalian cells. Together with *E. coli* TyrRS, it was used for incorporation of a number of UAAs in mammalian cells.[^23b^, ^24^]

Another more general approach that was shown to be useful for expressing bacterial/archaeal tRNAs in mammalian cells was developed in 2007.^[25]^ To overcome the problem of differences between bacterial and eukaryotic tRNA promoters, Wang et al. designed a strategy based on the type 3 eukaryotic pol III promoters, which include H1, U6 and 7SK.^[22]^ These promoters have no intragenic sequences. Thus, it is possible to express prokaryotic tRNAs in eukaryotic cells by placing such promoters upstream of the tRNA gene. With this approach, the *E. coli* aaRS/tRNA pair was introduced in mammalian cells and primary neurons with the help of H1 promoter.^[25]^ A later study with the PylRS/tRNA pair showed that U6 promoter can be used as well.^[18b]^ Since then, type 3 pol III promoters have proved to be the most robust approach available for orthogonal tRNA expression in eukaryotes.

## 3 Applications of Genetic Code Expansion for Structural Studies in Neurobiology

As mentioned, Wang et al. showed in 2007 that UAAs could be genetically encoded in different mammalian cells, including primary neurons.^[25]^ In the same article, they reported how UAA‐based mutagenesis could be used to gain unique insights into the dynamic processes of ion channel activation and inactivation. The authors studied fast N‐type inactivation of a voltage‐gated Kv1.4 channel by site‐directed mutagenesis.

**Figure 3:**
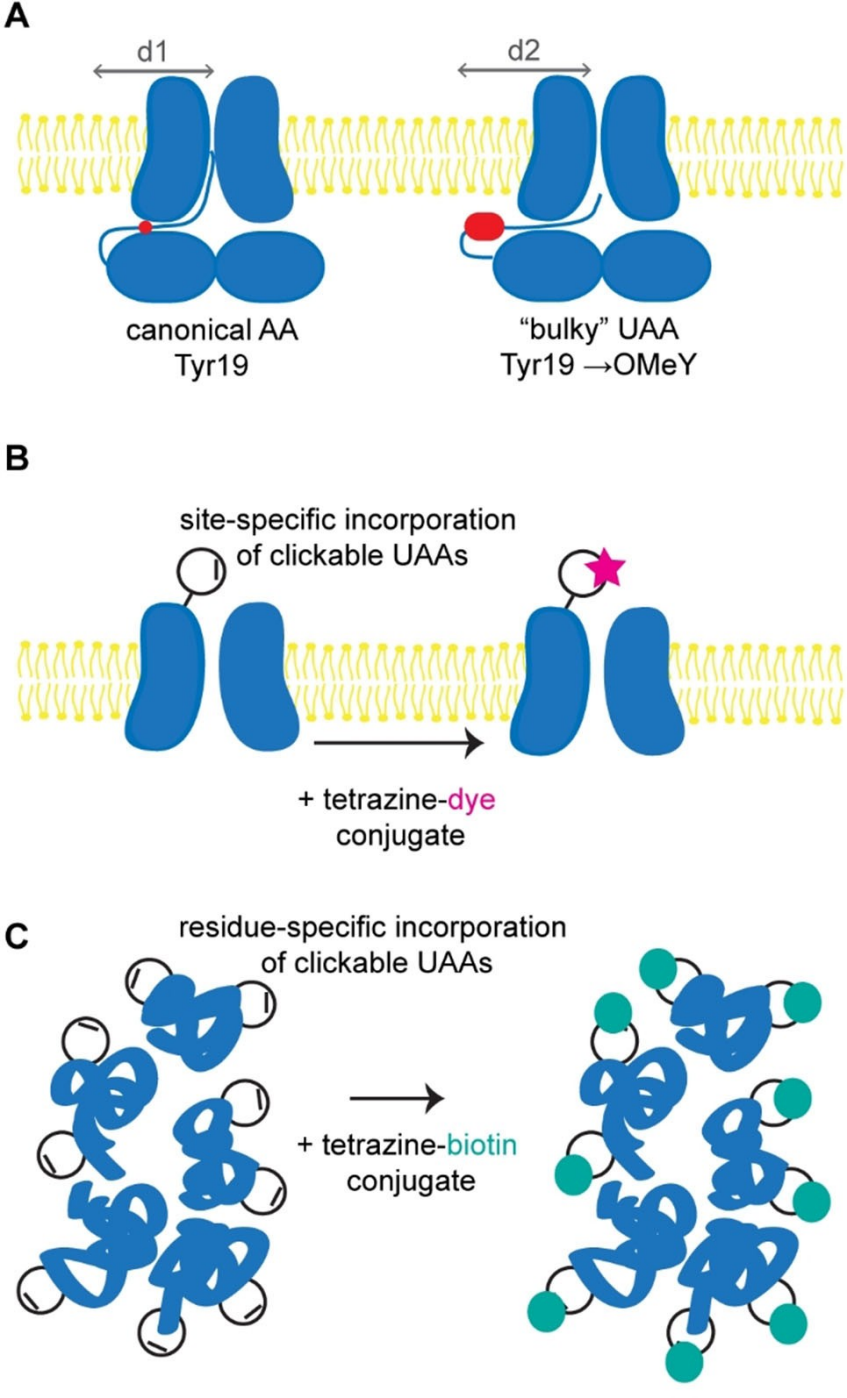
Some of the applications of UAAs in neurobiology. A) Structural studies with bulky OMeY, as described in ref. [25]. B) UAA‐based bioorthogonal fluorescent labelling for microscopy.[^32f^, ^35^, ^36^, ^37^, ^38^] C) click chemistry‐based pull‐down for tissue‐specific proteomics.[^32h^, ^54^]

By incorporating a bulky UAA with an extended side chain, OMeY, in the inactivation domain (Tyr19 position), they generated channels with markedly slower inactivation (Figure 3A). This suggests that the positioning of the inactivation peptide in the inner pore determines the rate of N‐type inactivation. Mutagenesis with canonical amino acids, Phe or Trp, had no effect on the channel inactivation. The reason for this is that in contrast to canonical amino acids, OMeY (Figure 2) lengthened the channel in the direction orthogonal to the peptide backbone. This prevented the N‐terminal inactivation peptide from extending into the cytoplasmic domain and increased the diameter of the peptide cross‐section (Figure 3A). Furthermore, with amber codon suppression the authors could test multiple individual positions and confirm that the abolition of N‐type inactivation is related to specific changes in the orientation of the inactivation peptide.

These results elegantly demonstrate the power of UAA‐based mutagenesis. The contribution of single amino acids to biological activity can be measured, and UAAs can introduce new properties compared to canonical amino acids. In the case of this study, UAAs introduced a physical constraint that gave a unique insight into the structure‐function relationships of Kv1.4.

## 4 Applications of Genetic Code Expansion‐based Bioorthogonal Labelling for Microscopy Studies

The combination of GCE and bioorthogonal chemical reactions has emerged as an important approach for protein labelling in vitro and in vivo. UAAs with reactive handles enable conjugation of target proteins with different probes, such as fluorescent dyes,^[26]^ tracers for positron emission tomography and magnetic resonance imaging,^[27]^ probes for protein pull‐down and enrichment.[^9a^, ^28^] UAA‐based conjugation chemistries have been described in detail in recent reviews.^[26]^ Therefore, the aim in this Minireview is not to be comprehensive, but rather to summarise the most recent studies with GCE and UAAs for bioorthogonal labelling in living mammalian cells, with a focus on fluorescent labelling and microscopy applications.

Protein labelling in living mammalian cells requires bioorthogonal and fast reactions that proceed at physiological conditions (i. e., an aqueous and catalyst‐free environment at physiological temperature). Freeing alkyne‐azide cycloaddition of its demand for a copper(I) catalyst has afforded new possibilities for labelling biomolecules in living cells and organisms.^[29]^ Strained alkynes can be conjugated with azides in a strain‐promoted alkyne‐azide cycloaddition (SPAAC) reaction. Another type of bioorthogonal reaction that is gaining a lot of interest for labelling in living systems is strain‐promoted inverse‐electron‐demand Diels‐Alder cycloaddition (SPIEDAC).^[30]^ In SPIEDAC, strained alkynes or alkenes react with 1,2,4,5‐tetrazines. SPAAC and SPIEDAC are frequently referred to as copper‐free click chemistry reactions. As discussed by Jewett et al., this relies on a broader definition of click chemistry to include reactions “that meet the necessary criteria of being selective, high yielding, and having good reaction kinetics”.^[31]^

UAA‐based protein labelling is a two‐step process. The first step involves co‐translational introduction of the reactive handle, in the form of a UAA, into the target protein. This is achieved by GCE. The second step is post‐translational labelling with a bioorthogonal click reaction of choice (Figure 3B). To date, UAAs carrying azide and tetrazine handles, as well as strained alkyne and alkene derivatives of l‐lysine, such as cyclooctyne (SCO‐K), bicyclononyne (BCN‐K), cyclopropene (Cp‐K) and *trans*‐cyclooctene (*trans*‐cyclooct‐4‐enyl, TCO‐K; *trans*‐cyclooct‐2‐enyl, TCO*‐K) derivatives (Figure 2),^[32]^ have been genetically encoded in *E. coli* and mammalian cells, and used for SPAAC/SPIEDAC‐based protein bioconjugation.

SPAAC and SPIEDAC reactions of azide and tetrazine dye derivatives with “clickable” UAA residues carrying strained alkynes and alkenes[^32a^, ^32b^, ^32c^, ^32d^, ^32f^] are often fluorogenic, a useful feature for applications in microscopy. The electronic properties of azides and tetrazines can quench the fluorescence of certain fluorophores,^[33]^ meaning that free (unincorporated) dye molecules are virtually dark and the fluorescence is restored only in the labelled protein product.

In recent years, SPIEDAC reaction is garnering more interest for protein labelling in microscopy than SPAAC because of its reactivity (SPIEDAC is orders of magnitude faster than SPAAC, with reaction rates >10^5^ m ^−1^ s^−1^ for SPIEDAC and <1 m ^−1^ s^−1^ for SPAAC). As only one amino acid is exchanged for the UAA which is subsequently directly reacted with a fluorescent dye, SPIEDAC‐based labelling tags provide the steric advantage of bringing the dye as close as possible to the protein of interest. This is especially useful for super‐resolution microscopy where larger labelling tags can introduce artefacts.^[34]^ To date, SPIEDAC between genetically encoded TCO*‐K, TCO‐K, BCN‐K, SCO‐K and tetrazine dye derivatives was applied in super‐resolution imaging of extracellular and intracellular proteins, such as insulin and EGF receptors, cytoskeletal proteins, and nucleoporins.[^32f^, ^35^] GCE and click chemistry have also recently been applied to the fluorescent labelling and imaging of neuronal proteins in mammalian cells. Neubert et al. site‐specifically labelled the extracellular domain of the NR1 (also known as GluN1) subunit of the NMDA glutamate receptor with SPIEDAC between genetically encoded TCO*‐K and Cy5‐tetrazine.^[36]^ Conventional immuno‐cytochemistry with antibodies resulted in an inhomogeneous (spotted) pattern, whereas SPIEDAC labelling of the NMDA receptor gave homogenous membrane staining when imaged with super‐resolution microscopy.^[36]^ In another study, BCN‐K was genetically encoded in Shaker B voltage‐dependent potassium channel.^[37]^ Labelling with Cy3‐tetrazine was achieved either site‐specifically at the position 345 in Shaker B channel or by labelling the 14‐residue long linker that was introduced between residues 344 and 345. UAA‐based labelling can also be combined with other labelling tags. For example, SPIEDAC labelling and the SNAP tag were combined to establish a method for the real‐time monitoring of γ‐secretase cleavage of amyloid‐β protein precursor (AβPP).^[38]^

However, despite the successful application of SPIEDAC reactions between a genetically incorporated TCO*‐K and a tetrazine dye derivative for fluorescent labelling,[^32f^, ^35b^, ^36^, ^38^] one potential drawback of such reactions is that under certain conditions the reaction product can undergo β‐elimination to release the target protein (as the lysine form) and a fluorescent artefact.^[39]^ Although this is useful for drug release^[39b]^ and decaging^[39c]^ studies, one can envisage problems in microscopy studies in which the fluorescent artefact might compromise accurate imaging. Thus, depending on the application, clickable UAAs based on bicyclononyne[^32c^, ^35a^] or more stable *trans*‐cyclooctene derivatives can be used.^[40]^

In addition to UAAs with reactive handles for bioorthogonal labelling, UAAs bearing fluorophores in their side chains can be directly incorporated into target proteins with GCE. The advantage of this approach is that such UAA residues do not need to be labelled post‐translationally. This overcomes the problems of dye delivery across the cell membrane and background intracellular labelling, but suffers the drawback that structurally more complex fluorophores that are particularly suitable for super‐resolution microscopy (e. g., Cy5, Alexa Fluor 647), cannot be incorporated into target proteins during protein translation owing to steric reasons. In this case, post‐translational labelling as described above is a more suitable strategy.

However, fluorescent UAAs have other useful properties that have been exploited for neurobiology studies. For example, 2‐amino‐3‐[5‐(dimethylamino)napththalene‐1‐sulfonamido]propanoic acid (dansyl‐alanine, Figure 2)^[41]^ was incorporated into the voltage‐sensitive domain (VSD) of *Ciona intestinalis* voltage‐sensitive phosphatase to study membrane depolarisation in neuronal stem cells.^[42]^ The dansyl fluorophore is sensitive to changes in environment polarity: if the polarity of its environment decreases, its fluorescence intensity increases. Upon membrane depolarisation, the VSD undergoes conformational change, which in this study, was accompanied by a change in fluorescence intensity.^[42]^ This approach could be used to study conformational changes of other membrane‐potential‐sensitive proteins, such as ion channels.

## 5 Application of Genetic Code Expansion to Site‐Specific Optogenetics in Neurobiology

**Figure 4:**
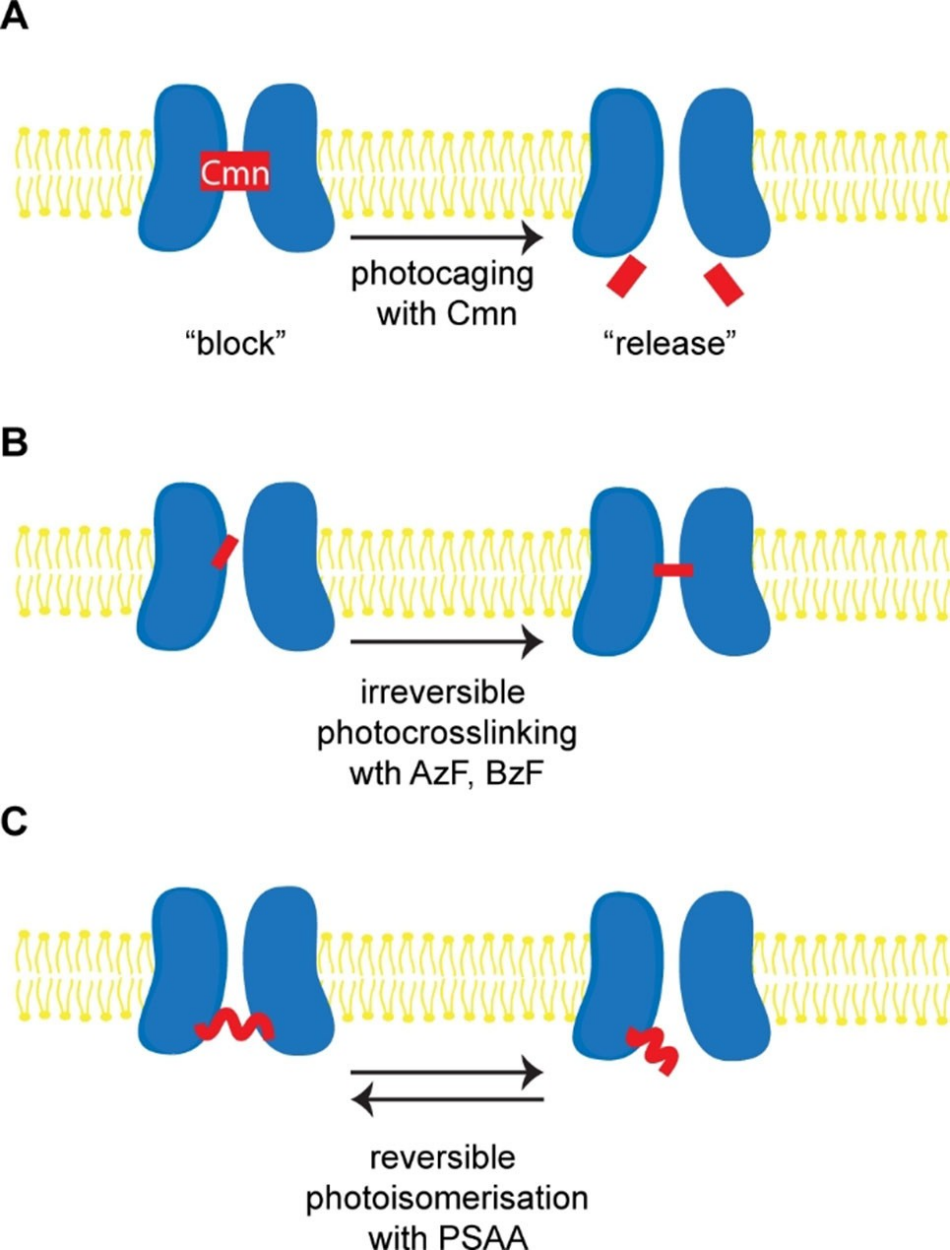
Some of the applications of UAAs in neurobiology. A) UAA‐based optogenetic studies with photocages.^[45]^ B) Irreversible photo‐crosslinking.[^46^, ^47^] C) Reversible photo‐isomerisation.^[52]^

Optogenetic methods using light to control biological processes are among the most widely used techniques for studying neuronal activity.^[3]^ By genetically encoding light‐sensitive ion channels, such as channelrhodopsin and halorhodopsin, in cells of interest, light can be used to switch on or off specific cell types to help decipher their role in complex neuronal networks. However, this approach relies on exogenous light‐sensitive proteins, which can be derived from algae, fungi or other microorganisms. The question is, can we engineer light sensitivity into native proteins? In addition to a plethora of photopharmatological compounds,^[43]^ photo‐crosslinkers, photocaged and reversibly photo‐responsive UAAs can be used to engineer light sensitivity into target proteins^[44]^ (Figure 4).

The first application of UAA‐based optogenetics in neurons in vitro and in vivo was published in 2013. Kang et al. created a light‐activated switch for suppressing neuronal firing by incorporating a photoreactive UAA into the Kir2.1 channel.^[45]^ Kir2.1 is an inwardly rectifying potassium channel, mainly expressed in skeletal muscle, brain and heart. It regulates neuron excitability, action potential cessation, heart rate, salt balance and hormone secretion. To control this protein with light, 4,5‐dimethoxy‐2‐nitrobenzyl‐cysteine (Cmn, Figure 2) was incorporated site‐specifically (Figure 4A). Before photoactivation, Cmn acts as a photocage and occludes the Kir2.1 pore, making the channel nonconductant. After brief UV light illumination, the nitrobenzyl group is cleaved and the channel opens. In this way, Kang et al. had demonstrated a photoinducible inwardly rectifying potassium (PIRK) channel in rat hippocampal neurons in vitro.

To make full use of PIRK channels, Kang et al. also managed to express them in embryonic mouse neocortex in vivo. This was achieved by in utero electroporation, and although the expression efficiency was low, this was the first example of GCE in living mammals. This study affords new opportunities for the field of molecular neuroscience, and optogenetics in general. Moreover, this approach can be used for other channels, receptors and signalling proteins in the brain. What makes it attractive is that upon removal of the photocaging group, a canonical residue is generated (cysteine in the case of Cmn). As a consequence, any Cys‐tolerant positions can be modified in these types of experiments.

In addition to photocaged UAAs such as Cmn, photo‐crosslinking UAAs have been employed for optogenetic experiments in neurobiology (Figure 4B). For example, *p*‐benzoyl‐l‐phenylalanine (BzF, also known as Bpa, Figure 2) and *p*‐azido‐l‐phenylalanine (AzF, Figure 2) were used to study AMPA glutamate receptors.^[46]^ Klippenstein et al. achieved selective and potent UV‐driven photo‐inactivation of homomeric (GluA2) and heteromeric (GluA2:A1) AMPA receptors.^[46a]^ The receptors could be rapidly inactivated within a period of just 10 s. This is much faster compared to other methods of inactivation such as RNA interference or the use of 6‐azido‐7‐nitro‐1,4‐dihydroquinoxaline‐2,3‐dione (ANQX), which can take hours to days. Poulsen et al. used UAA‐based mutagenesis to study conformational changes in the AMPA receptor pore domain during gating.^[46b]^

Zhu et al. achieved light control of an NMDA ionotropic glutamate receptor (NMDAR) by genetically encoding AzF.^[47]^ NMDARs are heterotetramers composed of two glycine‐binding (GluN1) and two glutamate‐binding (GluN2) subunits. GluN1 is an obligatory subunit, encoded by a single gene, whereas there are four types of GluN2 subunits (GluN2A–D). AzF was incorporated into the GluN1 subunit. Photo‐crosslinking of AzF with either the GluN2A or the GluN2B subunit revealed differences between subunit interactions. This approach could be used to understand the differences between different subtypes of NMDAR, such as subunit‐selective binding of certain allosteric modulators. In another study, allosteric regulation of an NMDAR was studied with AzF.^[48]^ These experiments were not performed in mammalian cells, but in *Xenopus* oocytes. However, instead of being microinjected with chemically acylated tRNAs, oocytes were transfected with an orthogonal aaRS/tRNA pair. This yielded enough protein to study allosteric regulation with ifenprodil and zinc.

One problem with photo‐crosslinkers, such as AzF and BzF, is that the changes they induce are not reversible. To achieve reversible photocontrol of target proteins, the azobenzene group has been employed. The azobenzene group undergoes wavelength‐dependent reversible *trans*‐*cis* isomerisation^[49]^ and has formed the basis for the design of photo‐switchable diffusible and tethered ligands for neuroreceptors and ion channels.^[50]^ However, such ligands are restricted to solvent‐accessible (usually extracellular) sites and to the methods of conventional protein bioconjugation. To gain site‐specific control of any domain and position in a protein of interest, the photo‐switchable azobenzene group should be incorporated at those positions directly.^[51]^ Klippenstein et al. achieved fast and reversible control over a set of NMDAR subunits incorporating an azobenzene‐based photo‐switchable UAA (PSAA, Figures 2 and 4C).^[52]^ Modifying different positions allowed the authors to either inhibit or potentiate the activity of NMDAR, thus illustrating the power of site‐specific UAA incorporation. Notably, the optical control of a protein‘s function with high temporal resolution and molecular specificity can be reversibly mediated by *cis*‐*trans* isomerisation of a single amino acid side chain. However, to realise the full potential of this technology, it is necessary to move it to the level of whole animals, which the next section of this Minireview addresses.

## 6 Genetic Code Expansion in Mouse Brain: Current Status

**Figure 5:**
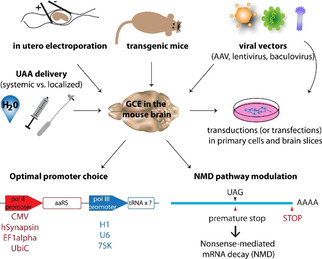
Overview of the components necessary for successful genetic code expansion (GCE) in the brain or primary neurons. Individual factors, such as optimal vectors, optimal promoters, UAA delivery, transgenic versus transient transfections are discussed in Sections 6 and 7. The image of the mouse brain is provided by the Database Center for Life Science (DBCLS).

One of the greatest remaining challenges towards the wide‐spread application of GCE in physiological studies is to further optimise it at the whole‐animal level.[^4c^, ^4d^] The mouse is a particularly useful model organism for neurobiology studies and much effort has been put into expanding its genetic code (Figure 5).

The first example of UAA incorporation in a living brain was published in 2013.^[45]^ In this study, Kang et al. generated a light‐activated switch for controlling Kir2.1 protein. To achieve this, an orthogonal aaRS/tRNA pair was introduced into embryonic mouse brain with in utero electroporation. This was also the first example of an expanded genetic code of a living mammal. However, as we discussed in the previous chapter, in utero electroporation does not provide high UAA incorporation efficiency.

More recently, Ernst et al. developed adeno‐associated virus (AAV) vectors for GCE in mice.^[53]^ In this proof‐of‐principle study, the PylRS/tRNA pair was expressed in the live adult brain and used to incorporate UAAs site‐specifically into GFP as a reporter protein by amber codon suppression. In another study, the PylRS/tRNA pair was expressed by stereotactic injections of AAV vectors into different regions of the brain in living mice for cell‐specific proteomic studies.^[54]^ This approach is called stochastic orthogonal recoding of translation (SORT) and was first developed in *Drosophila melanogaster*.^[32h]^ SORT is not based on amber codon suppression, but on reassigning one of the sense codons to incorporate the UAA. By genetically targeting the PylRS/tRNA pair to the cells of interest, whole proteomes from specific cells can be tagged with the clickable UAA (Figure 3C). Subsequently, tagged proteomes can be specifically labelled (SORTM) or pulled‐down (enriched) and identified (SORT‐E) with click chemistry. SORT nicely complements the metabolic labelling approach for the enrichment of cell‐specific nascent proteomes in mice that was recently applied to identify changes in the de novo proteome during memory formation in the hippocampus^[55]^

In addition to AAVs, lentiviruses have been used for GCE.^[42]^ However, similarly to AAV vectors, they might not be optimal because of the risk of recombination on incorporating repetitive sequences, such as multiple orthogonal tRNA gene copies. As an alternative, PiggyBac transposon‐mediated integration can be used. This method was applied for stable integration of the PylRS/tRNA pair in a mammalian genome.^[56]^ PiggyBac allows for multiple copies of tRNA to be stably expressed, which is advantageous when optimising the efficiency of GCE. Although so far only used for GCE in cellulo, PiggyBac technology could be used to generate transgenic mice.^[57]^ To establish stable GCE in mammalian cells, a recent study developed Epstein‐Barr virus‐based episomal vector to encode UAAs in human hematopoietic stem cells and their differentiated progenies.^[6]^ Engineered cells were successfully engrafted in mice, offering a possibility for generation of humanised mice models with expanded genetic code.

An alternative method for GCE in brain tissue is the use of baculovirus vectors, as reported by Zheng et al.^[58]^ Baculoviruses have large cargo capacity and are more resistant to the presence of repetitive sequences in their genomes. Consequently, they might be more suitable for the expression of multiple tRNA gene copies. Zheng's study with baculovirus vectors was performed on brain slices, and not whole animals, but it revealed an interesting finding with regards to the optimal vector design for GCE.

Based on their previous work,^[59]^ Zheng et al. made an improved baculovirus vector for GCE in mammalian cells. In contrast to transient transfections, transductions with baculovirus vectors showed a linear increase in the GFP reporter levels with an increasing number of transduced baculovirus particles. That allowed systematic investigation of the individual contribution of the orthogonal tRNA and aaRS on the expression of the reporter gene. It had been noted earlier that low tRNA levels might be a limiting factor, leading to a low efficiency of UAA incorporation with GCE. The solution to this is to increase the levels of tRNA by increasing the number of tRNA copies.[^24b^, ^60^] Interestingly, Zhang et al. discovered that it is not only the absolute numbers that need to be taken into account, but that a suboptimal ratio of tRNA to aaRS can also lead to low efficiency of amber codon suppression. More specifically, they noted that under the conditions of limited tRNA, high levels of aaRS led to a reduction in levels of the reporter gene. Most likely, overexpressed aaRS sequesters tRNA molecules and thus reduces the efficiency of amber codon suppression. Consequently, in addition to increasing the number of tRNA copies, it was necessary to lower the amount of aaRS. To achieve this, instead of using strong promoters, such as CMV, EF1alpha or human synapsin promoter,[^53^, ^61^] the authors tested three weaker promoters for aaRS expression: SV40, human PGK and UbiC. Their improved baculovirus vector (named pacbac3) with the UbiC promoter and 16 tRNA copies (expressed under the H1 promoter) was used for the efficient incorporation of OMeY, AzF and *p*‐acetyl‐l‐phenylalanine (pAcF) in the brain slices. Amber mutant GFP reporter levels were similar to those of the wild‐type GFP reporter, which was not the case with other versions of tested baculovirus vectors or with transient transfections.

As an alternative to viral transductions and PiggyBac transposons, two transgenic mouse lines for the GCE were developed in 2017.[^61^, ^62^] Han et al. generated a transgenic mouse for the incorporation of *N*‐acetyl‐lysine (AcK, Figure 2).^[61]^ Chen et al. reported on the generation of a transgenic mouse and zebrafish for the incorporation of AzF.^[62]^ To make amber codon suppression heritable, Han et al. made two transgenic lines: an AcKRS mouse, stably expressing a pyrrolysine‐derived AcK‐specific aaRS/tRNA pair, and a GFP(TAG) mouse, stably expressing an amber mutant of a reporter protein, GFP. By crossing these two lines, a double transgenic heterozygous AcKRS‐GFP(TAG) mouse was created. The authors achieved spatiotemporal control of site‐specific acetylation by injecting the AcK at a specific time point and into selected tissue. They showed evidence of successful AcK incorporation in a number of different tissues. However, although they showed with RT‐PCR that transgenes (GFP(TAG) and AcKRS) were stably integrated in the brain, no evidence of successful AcK incorporation at the protein level in the brain was provided. By contrast, in the study of Chen et al., only the AzFRS/tRNA pair was heritable. To achieve amber codon suppression, primary cells were isolated from the transgenic mouse and transfected with an amber mutant of the GFP construct. Because the protein of interest needs to be applied exogenously, the utility of the reported transgenic mouse might be limited for whole‐animal experiments, but is of advantage for studies with primary mouse cells.

## 7 Challenges Associated with Genetic Code Expansion and Outlook

GCE in living cells has emerged as a powerful method to probe and manipulate protein structure and function. The studies summarised in this Minireview exemplify the application of GCE in neurobiology and some of the factors critical to its successful implementation (Figure 5). Most of these factors are important not only for in vivo experiments, but also in cell culture. However, with more complex biological systems, such as animal models, new challenges emerge.

### 7.1 Efficiency of amber codon suppression

One of the principal challenges of optimising GCE for biological studies is to increase the efficiency of site‐specific UAA incorporation with amber codon suppression. Efficiency can be improved with optimal vector design and good choice of promoters (Figure 5). This is especially relevant for experiments with new host cells and animal models. On the other hand, residue‐specific incorporation of UAAs for tissue‐specific proteome labelling,[^32h^, ^54^] might benefit from the lower incorporation efficiency because of the potential toxic effects of proteome‐wide incorporation. Another phenomenon that affects the efficiency of amber codon suppression is the nonsense‐mediated decay (NMD) pathway (Figure 5). NMD is a surveillance pathway present in all eukaryotes, which serves to protect cells from genetic mutations by degrading mRNAs that contain premature stop codons. This is a problem for GCE technology because we deliberately introduce amber stop codons into genes of interest to achieve site‐specific incorporation of UAAs. Studies in *Caenorhabditis elegans* and fibroblasts derived from AcKRS/tRNA transgenic mice showed that downregulation of the NMD leads to higher UAA incorporation rates.[^61^, ^63^]

The context of the amber codon is also important for successful amber suppression. It seems likely this affects the NMD pathway and the general stability of mRNA molecules; a recent review summarises what little we know about this.^[64]^ Another limiting factor is the competition between orthogonal tRNA and the translation termination machinery that might recognise premature stop codons. In accordance with this, experiments with engineered eukaryotic translation termination factor 1 show increased amber codon suppression.^[60]^

### 7.2 tRNA stability and aaRS/tRNA ratio

In addition to mRNA stability, the availability and stability of the tRNA can affect GCE efficiency. Recent studies have focused on making more‐stable, rationally designed tRNAs for the PylRS/tRNA system.^[65]^ Furthermore, whereas most researchers would argue that more tRNA is better, one study reported that too much tRNA can lead to problems, such as background labelling in fluorescence microscopy studies.^[66]^ This means that depending on the application, it is necessary to determine the optimal amount of both tRNA and aaRS. As discussed earlier, the ratio between the tRNA and aaRS also needs to be taken into account,^[58]^ otherwise, tRNA might be sequestered by the aaRS. Indeed, a recent study showed that PylRS can sequester its cognate tRNA^Pyl^.^[35b]^ Unexpectedly, both PylRS and tRNA^Pyl^ were trapped in the nucleus instead of the cytoplasm where protein translation takes place. This was caused by the intrinsic nuclear localisation signal (NLS) found in the PylRS. By fusing the PylRS to the strong nuclear export signal (NES), both ^NES^PylRS and tRNA^Pyl^ were preferentially located in the cytoplasm. This led to increased efficiency of amber codon suppression.

### 7.3 UAA delivery

Another aspect crucial for successful GCE is delivery of the UAA. This is especially relevant when working with the nervous system in vivo, because the UAA needs to cross the blood‐brain barrier (Figure 5). In the pioneering study by Kang et al,^[45]^ Cmn was injected directly into the lateral ventricle of the mouse brain. To increase the bioavailability of the UAA, the dipeptide Cmn‐alanine was synthesised, an approach that had previously been successful for increasing uptake of Cmn in *C. elegans*.^[67]^ UAAs can also be provided to animals in drinking water,[^53^, ^54^, ^55^] with the use of osmotic pumps and intracerebroventricular catheters,^[53]^ or by intraperitoneal^[61]^ injection. Han et al. performed daily intraperitoneal injections, but as mentioned above, they did not show evidence of AcK incorporation in the brain, so it remains unknown if the AcK crossed the blood‐brain barrier.^[61]^ This is a problem that likely needs to be addressed for individual UAAs because they have different physicochemical properties affecting their uptake and distribution. In determining the most optimal mode of UAA delivery, cost of the UAA also needs to be taken into account; the large amount of UAA required for experiments in animals can be expensive. As a result, UAA delivery with stereotactic injections or osmotic pumps might be more suitable than peroral, intraperitoneal or intravenous administration.

### 7.4 Potential toxicity

Ernst et al. showed that the physiological functions of neurons, crucial for the circadian circuit, were not perturbed by incorporation of the UAA.^[53]^ In addition, previously developed transgenic animals such as *D. melanogaster*,^[68]^ *C. elegans* ^[63]^ and mice^[54]^ showed no obvious developmental or growth defects. However, in trying to increase the efficiency of amber codon suppression, it is necessary to think about how its potentially toxic effects can be avoided. One of the most pertinent questions is what happens with other native amber codons? Initially, the amber codon was chosen because in bacteria only 8–9 % of genes contain an amber stop codon. In eukaryotes, this proportion is around 20 %, which might pose a problem. Until now, this was studied only in bacteria.^[69]^ It seems that in the presence of translation termination factor RF1, there was no effect on the growth rate of *E. coli* and naturally occurring stop codons were unaffected. However, modifying RF1 lowered the growth rate of *E. coli*.^[70]^ By using unique quadruplet codons^[12d]^ or unnatural codons,^[71]^ some of these problems can be avoided owing to the lack of competition with native amber codons. A further promising strategy for avoiding interference with the host is based on a parallel genetic code utilising engineered orthogonal ribosomes.^[72]^ Orthogonal ribosomes are unnatural ribosomes that are directed towards orthogonal mRNAs in *E. coli*. Their application leads to increased efficiency of amber codon suppression, probably by avoiding interference with RF1.^[73]^ They can even be combined with quadruplet codons for incorporation of multiple distinct UAAs.[^20d^, ^74^] However, implementing strategies such as orthogonal ribosomes and unnatural codons for efficient GCE at the eukaryotic (mammalian cell) level is not straightforward.

Another recently developed approach for GCE in mammalian cells involves artificial membrane‐less organelles designed for amber codon suppression.^[75]^ This approach enables orthogonal translation in a specialised organelle. This minimises the effect on the host, as amber codon suppression happens exclusively in the selected mRNAs that are targeted to this organelle.

### 7.5 Outlook

GCE and UAAs with diverse side chains enable a development of novel approaches for the manipulation of biological processes with site‐specific or residue‐specific precision. In this Minireview, the use of GCE technology in neurobiology was discussed. A wide range of UAA‐based structural, labelling and optogenetic studies of neuronal proteins nicely illustrates the power and potential of this technology.

Although more at the proof‐of‐principle level for now, GCE in living cells and animals has the potential to provide novel insights into molecular neurobiology, by complementing existing techniques and addressing scientific problems that are not readily addressed otherwise. In this regard, particularly relevant are the steric advantages offered by GCE‐based site‐specific protein manipulation with UAAs. Incorporation of UAAs with amber stop codon suppression is not only site‐specific, but also minimally invasive as only one amino acid is exchanged for a clickable, photo‐switchable, or other type, of UAA.

Minimal fluorescent labelling tags based on clickable UAAs are particularly beneficial for super‐resolution microscopy.^[34]^ Fluorescent dyes with optimal photophysical properties can be attached in living cells with site‐specific precision directly to target proteins; at present, this cannot be achieved with any other method. The steric advantages that accompany small tag size and high labelling density are essential for optimal resolution.[^34b^, ^76^] In addition, for fluorescent labelling of certain proteins, the size of the labelling tag is of critical relevance because larger labels such as fluorescent protein fusions can introduce artefacts.^[77]^ Owing to their complex structure and function, neuronal proteins such as ion channels and receptors, are especially sensitive to modifications. Furthermore, ultrafast bioorthogonal SPIEDAC reactions are compatible with living cells and could be combined with state‐of‐the‐art live‐cell super‐resolution microscopy. Similarly, photo‐switchable UAAs expand the field of optogenetics by allowing us to control individual domains of proteins with light in a site‐specific manner, nicely complementing studies with photo‐switchable ligands.[^43^, ^50a^, ^50c^] Thanks to the developments in this field, opto‐proteomics^[78]^ has recently emerged as a term alongside optogenetics. Finally, switching from site‐specific to residue‐specific UAA incorporation facilitates new approaches to assess changes in cell‐ and tissue‐specific proteomes, a challenge that is not readily tackled with other methods.

As the field of GCE matures and becomes more suitable for studies involving animal models, we can expect more exciting findings and physiological applications of this technology. Furthermore, by designing orthogonal ribosomes^[72]^ and organelles,^[75]^ using unnatural codons^[71]^ and even modifying cells to biosynthesise amino acids bearing bioorthogonal handles,^[79]^ we are nearing the goal of a truly orthogonal GCE system. Achieving this goal would allow us to investigate and visualise biological processes without interfering with the host. This is relevant not only for neurobiology studies but for the molecular life sciences in general.

## Conflict of interest

The authors declare no conflict of interest.
